# Association between endometriosis and arthritis: results from NHANES 1999-2006, genetic correlation analysis, and Mendelian randomization study

**DOI:** 10.3389/fimmu.2024.1424648

**Published:** 2024-07-29

**Authors:** Huanying Xu, Haoxi Zou, Qidan Wen, Xiaoyan Xing, Ningning Xu, Suzhen Wu

**Affiliations:** ^1^ Foshan Clinical Medical School of Guangzhou University of Chinese Medicine, Foshan, Guangdong, China; ^2^ TCM Gynecology Department, Foshan Fosun Chancheng Hospital, Foshan, Guangdong, China

**Keywords:** endometriosis, arthritis, NHANES, genetic correlation analysis, Mendelian randomization

## Abstract

**Background:**

Previous studies reported that endometriosis may have a higher risk of arthritis. However, it remains unclear whether the association between endometriosis and arthritis has genetic correlations, or the relationship is causal. Linkage Disequilibrium Score (LDSC) and Mendelian Randomization (MR) analyses use genetic variation as a natural experiment to explore genetic correlations and causal inferences from observational data, reducing unmeasured confounding factors.

**Method:**

Participants (aged 20-54 years, n = 2,915) for the cross-sectional study were obtained from the National Health and Nutrition Examination Survey (NHANES). Endometriosis and arthritis were diagnosed based on self-reported by reproductive health and medical condition questionnaire. Weighted multivariable logistic regression was used to explore the relationship between endometriosis and arthritis. LDSC and MR analysis were performed using the genome-wide association study (GWAS) summary statistics to identify the causal association.

**Result:**

A significant positive association between endometriosis and arthritis was found after multivariable adjustment (OR = 1.89; 95% CI: 1.33, 2.67). When exploring different types of arthritis, a positive association was revealed with rheumatoid arthritis (RA), other types of arthritis, and cases that the arthritis type were unknown, with an OR of 2.07 (95% CI: 1.03, 4.17), 2.78 (95% CI: 1.30, 5.95), and 2.06 (95% CI: 1.36, 3.11), respectively. However, genetic correlation analysis between endometriosis and RA did not reveal any significant findings (all P values > 0.05). Moreover, MR analysis also failed to identify a causal relationship between endometriosis and RA (all P values > 0.05).

**Conclusion:**

Cross-sectional study identified a significant positive association between endometriosis and arthritis among US women, especially among RA, while findings based on LDSC and MR analysis did not support a genetic correlation or causal role. These findings suggest that clinicians should pay more attention to the coexistence of RA in endometriosis patients and explore the shared pathophysiological mechanisms of these two disorders, with a particular focus on extrinsic factors rather than intrinsic genetic inheritance.

## 1 Introduction

Endometriosis (EMs), characterized by the presence of endometrium-like tissue outside the uterus, is a chronic, inflammatory, and estrogen-dependent gynecologic condition known for its elusive cure, tendency to recur, and profound impact on women’s health and quality of life (1–3). It affects roughly 10% of reproductive-age women globally, estimated at around 190 million individuals based on World Bank data from 2017 (2). Endometriosis is associated with a wide range of symptoms, such as chronic pelvic pain, severe periods pain, painful sex, pain on defecation and urination, heavy menstruation, pelvic masses (chocolate cysts or pelvic nodules), and infertility (1). Epidemiological studies also report that women with endometriosis suffer from fatigue, depression, and seriously affect women’s mental health (4, 5).

Recent insights suggest that systemic chronic inflammation and enhanced oxidative stress were associated with the pathological mechanism of endometriosis (3, 6, 7). Considered as a systemic chronic disease, it’s crucial to be vigilant for comorbidities or subsequent disorders linked to endometriosis (1, 8), such as migraine (9), asthma (10), rheumatoid arthritis(RA) (11), systemic lupus erythematosus (8), cardiovascular disease (12), and even gynecologic cancers (13). Previous observational studies indicated that women with endometriosis may have a higher risk of arthritis than women without endometriosis (11, 14–16), particularly among the type of RA. For instance, the Nurses’ Health Study II (N = 114,453 women) over a 22-year follow-up period identified that endometriosis women confirmed by laparoscopically had a higher risk of RA than those without endometriosis (HR = 1.41; 95% confidence interval (CI): 1.05, 1.89) (11). Similarly, recent cohort studies conducted in various populations in Taiwan and Japan have also suggested an increased risk of RA among endometriosis women (16, 17).

These observational study findings had only found an association between endometriosis and arthritis, but whether this relationship is genetic, or causal remains unknown. In addition, some studies revealed that the co-occurrence of endometriosis and arthritis shared susceptibility loci and genetic polymorphisms (18). Confirmed by gene association studies and genome-wide association studies (GWAS), genetic polymorphisms related to both RA and endometriosis include the interleukin-6 (IL-6) gene, interleukin-10 (IL-10) gene, vascular endothelial growth factor gene, protein tyrosine phosphatase non-receptor type 22 gene, and signal transducer and activator of transcription 4 gene (18). However, whether these genetic polymorphisms have genetic correlations is unknown.

In recent years, statistical methods based on GWAS have been proposed to estimate the genetic correlation and causality between traits. Linkage Disequilibrium Score (LDSC) is a widely used method in genetic correlation analysis to estimate the heritability of complex diseases and traits (19). It leverages the concept of linkage disequilibrium (LD) by calculating the LD score for each single nucleotide polymorphism (SNP). The LD score measures the degree of correlation between a given SNP and its surrounding SNPs. By using these scores, LDSC can infer the strength of association between genetic variants and complex traits. However, the use of LDSC alone often provides only a genetic association between diseases and traits, leaving the causal relationship between the two traits unclear. Mendelian Randomization (MR) is an application of instrumental variable analysis used to evaluate causal relationships between exposure and outcome (20). In MR analysis, SNPs are commonly used as instrumental variables for the putative risk factor. Because genotype precedes phenotype and alleles are randomly assigned at conception, MR using genetic variation as an instrumental variable helps to avoid measurement bias, confounding bias, and reverse causality interference (21). Thus, MR is very convincing in verifying the causal relationship between exposure variables and outcome variables, especially when the two have been shown to be genetically correlated.

LDSC and MR analyses could further dissect the genetic correlations and causal relationships between endometriosis and RA. However, to our knowledge, few studies have been reported to investigate the relationship between endometriosis and arthritis using three study design methods: observational study, genetic correlation analysis, and MR study. Therefore, we comprehensively explore the association between endometriosis and arthritis, using the National Health and Nutrition Examination Survey (NHANES) 1999-2006 database, genetic correlation analysis, and MR analysis methods based on GWAS data. In this study, we first conducted an observational study using NHANES data and hypothesized that endometriosis is positively associated with arthritis. Secondly, we performed genetic correlation analyses and MR studies using the summary statistics from several large endometriosis and RA GWAS to identify any significant genetic correlations and causal relationships between endometriosis and RA, and hypothesized that endometriosis has genetic correlations and causal relationships with RA. These analyses will provide insights into the causal relationship and genetic background between endometriosis and arthritis, and improve our understanding of their underlying biology.

## 2 Methods

### 2.1 Study population in NHANES

The data was sourced from the NHANES database, which is publicly available on the official website (https://www.cdc.gov/nchs/nhanes/index.htm). The protocols of the NHANES study were authorized by the National Center for Health Statistics Research Ethics Review Committee (https://www.cdc.gov/nchs/nhanes/irba98.htm). All participants in the survey provided written informed consent.

**Figure 1:**
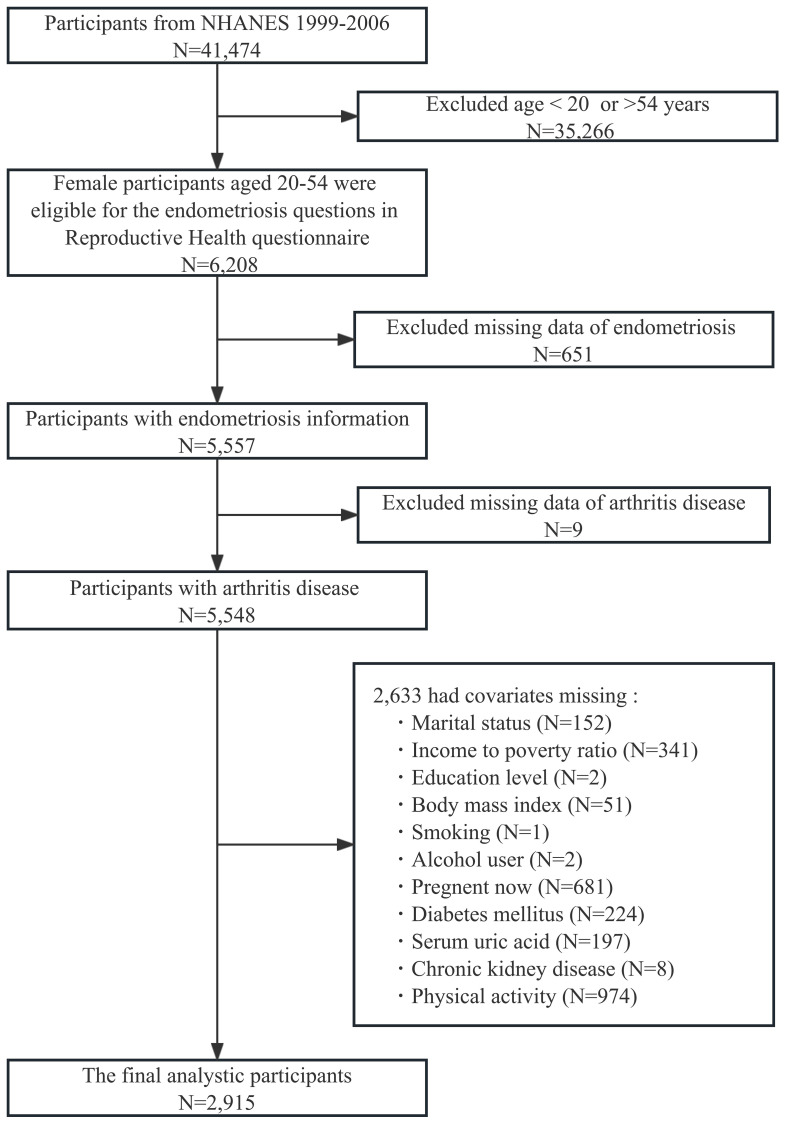
Flow chart of participants selection. NHANES, National Health and Nutrition Examination Survey.

This study utilized data from the NHANES 1999-2006 cycle with a total of 41,474 participants. We included female participants aged 20-54 years (N = 6,208), because participants within this age range were eligible for the endometriosis questions in the reproductive health questionnaire. Those with missing endometriosis information (N = 651), arthritis disease (N = 9), and other variables (N = 2,633) were excluded. Ultimately, 2,915 female participants were included in our analyses (shown in **Figure 1**).

### 2.2 Self-reported endometriosis and arthritis in NHANES

Self-reported endometriosis was diagnosed based on two questions from the reproductive health questionnaire: 1) RHQ360: “Has a doctor or other health professional ever told you that you have endometriosis?” and 2) RHQ370: “How old were you when you were first told had endometriosis?”. Any woman who self‐reported “Yes” to questionnaire RHQ360 or provided an answer to questionnaire RHQ370 was considered to have endometriosis.

The diagnosis of self-reported arthritis was established through three questions from the medical condition questionnaire: 1) MCQ160A: Has a doctor or other health professional ever told you that you had arthritis? 2) MCQ180A: Age when told you had arthritis? 3) MCQ190: Which type of arthritis? In NHANES 1999-2006 cycle, types of arthritis were divided into RA, osteoarthritis, other, and don’t know. Participants who answered “yes” to questionnaire MCQ160A or provided an answer to questionnaire MCQ180A or MCQ190 were considered to have arthritis.

### 2.3 Covariates in NHANES

To control potential confounding bias in this study, age, body mass index (BMI), race, marital status, education level, smoking status, alcohol status, income-to-poverty ratio (PIR), physical activity (PA), serum uric acid (SUA), chronic kidney disease (CKD), diabetes mellitus (DM), hyperlipidemia, and hypertension were selected as covariates based on clinical plausibility and previous research (16, 22–24). Age was divided into two groups based on clinical significance (<35, and ≥35 years). BMI was categorized into normal (<25 kg/m^2^), overweight (25-30 kg/m^2^), and obesity (≥30 kg/m^2^) by clinical significance. Smoking status was classified as “yes” or “no” based on self-reported having smoked at least 100 cigarettes in their lifetime. The categorization of alcohol status was determined through self-reporting as follows: heavy, self-reported ≥4 drinks every day; mild/moderate, self-reported ≤3 drinks every day; former, did not drink last year but drank ≥12 drinks in lifetime or self-reported ≥12 drinks in 1 year and did not drink last year; never, self-reported <12 drinks in lifetime. PIR is categorized into three degrees (<1.5, 1.5-3.5, and ≥ 3.5). PA was assessed using weekly physical activity participation information collected by the Global Physical Activity Questionnaire (25). CKD was diagnosed according to the estimated glomerular filtration rate (26). DM is diagnosed if any of the following two conditions are satisfied: 1) doctor told you that you have diabetes. 2) Taking antidiabetic medication. The definition of hyperlipidemia meets one of the following three criteria: 1) Triglyceride levels ≥ 150 mg/dL. 2) Total cholesterol levels ≥ 5.18 mmol/L; low-density lipoprotein levels ≥ 3.37 mmol/L; high-density lipoprotein levels < 1.3 mmol/L. 3) Use of lipid-lowering medication. Hypertension was diagnosed if any of the following three conditions are satisfied: 1) doctor told you that you have hypertension. 2) Taking antihypertensive medication. 3) as a mean blood pressure exceeding 140/90 mmHg for systolic pressure and diastolic pressure, respectively.

### 2.4 GWAS sources

Data for endometriosis were collected from GWAS summary data of FinnGen consortium release data, UK BIOBANK release data, and Jiang L et al. (PMID: 34737426) (27). We used GWAS meta-analysis to integrate the above three GWAS studies to increase the sample size, improve the statistical power, and more accurately identify the genetic factors of diseases or phenotypes (28). GWAS Meta-analysis was conducted for 1,217,311 SNPs under a fixed-effects model with inverse variance weighting for overall endometriosis population. Finally, a total of 1,168,438 SNPs were used for linkage disequilibrium score (LDSC) analysis. Summary statistics on RA used in the current study were obtained from five GWAS, downloaded from the Integrative Epidemiology Unit (IEU) OpenGWAS database (GWAS ID: ukb-a-105, ukb-b-9125, ukb-b-11874, ukb-b-M06), and FinnGen consortium release data. Details of the GWAS studies included in our analysis were shown in **Supplementary Table 1**.

### 2.5 Statistical analyses

Weighting method was used in this study according to the complex NHANES data analysis (https://www.cdc.gov/nchs/nhanes/index.htm). For categorical variables (race, marital status, education level, smoking status, alcohol status, CKD, DM, hyperlipidemia, and hypertension), frequency and percentage (after weighting) were presented, while for continuous variables (age, BMI, PIR, PA, and SUA), median and standard deviation (SD) were presented. Pearson’s chi-square tests and Kruskal-Wallis tests were used to compare categorical and continuous variables between endometriosis and without endometriosis women, respectively.

Multivariable logistic regression was used to explore the relationship between endometriosis and arthritis by controlling four models. Crude model was unadjusted. Model 1 was adjusted for Age (continuous) + BMI (continuous). Model 2 was adjusted for model 1 + Race (White, Black, Mexican, Hispanic, Other race), Marital status (Married/Living with partner, Never married, Divorced/Separated/Widowed), Education level (College graduate or above, Some college or AA degree, High school/GED/Less than 11th grade), Smoking status (No, Yes), Alcohol status (Never, Former, Mild/Moderate, Heavy), PIR (continuous), and PA (continuous). Model 3 was adjusted for model 2 + SUA (continuous), CKD (No, Yes) + DM (No, Yes) + Hyperlipidemia (No, Yes) + Hypertension (No, Yes). We further performed subgroup and interaction analyses by all covariates to ensure the robustness of the result.

To investigate the overall genetic relationship between endometriosis and RA, we performed LDSC analysis (19, 29). As for MR analysis, it needs to satisfy three assumptions in order to investigate causal effects of exposure on the outcome: 1) The genetic variants were supposed to be correlated with EMs; 2) They should not be associated with confounding factors; and 3) They should affect RA only as mediated by the EMs (30). Instrumental variables (IVs) were screened to satisfy these three major assumptions (31): 1) To fulfill the first hypothesis, we extracted SNPs associated with EM at the genome-wide significance level (p < 5e - 8) as instrumental variables and calculated the F-statistics of these SNPs to quantify the strength of the instrumental variables and to exclude weak instrumental variables (F-statistics < 10); 2) To fulfill the second hypothesis, SNPs in linkage disequilibrium were excluded (r^2^ threshold < 0.001 within a 10000 kb window), and the remaining SNPs were extracted from the outcome datasets; and 3) To fulfill the second and third hypotheses, we retrieved potential confounders (with a threshold of 5E-8) through Phenoscanner (http://www.phenoscanner.medschl.cam.ac.uk/) and excluded instrumental variables associated with confounders. Genetic variants that preferentially satisfied those assumptions were shown in **Supplementary Table 2**. Genetic associations with all exposures were taken from a meta-analysis of GWAS, we obtained SNP-specific Wald estimates and then used inverse variance weighting (IVW) with multiplicative random effects, MR-Egger, and weighted median (WM) (32). The IVW method is a classical method for MR analysis, where the weighted average is calculated by taking the reciprocal of the variance of each IV as the weight, ensuring the effectiveness of all IVs (20). MR Egger utilizes a weighted linear regression analysis, providing robust estimates that are independent of the validity of instrumental variables. Nevertheless, it is crucial to acknowledge that these estimates may have lower statistical precision and can be influenced by outlier genetic variation. On the other hand, The problem of estimation accuracy variability is tackled by the WM approach. In a manner reminiscent of the IVW approach, the WM method assigns inverse weights that are contingent upon the variance of individual genetic variants, demonstrating reliability even when causal effects are violated (33). In addition, two MR analysis methods, including simple mode and weighted mode, were used as supplementary analyses (33).

R software (version 4.3.2; http://www.R-project.org) was used to manage and analyze the data. A two-tailed P value of <0.05 was regarded as statistical significance.

## 3 Results

### 3.1 Characteristics of study population from NHANES

Characteristics of study population with weighted estimates are shown in **Table 1**. A total of 2,915 women aged 20-54 years from NHANES 1999-2006 were included, among whom 243 were told as having endometriosis. The mean age for women with endometriosis was 41.11 ± 0.49 years, while for those without endometriosis, it was 37.89 ± 0.27 years. In comparison to women without endometriosis, those with endometriosis were more likely to be Non-Hispanic White, Divorced/Separated/Widowed, with higher income-to-poverty ratio, and higher SUA levels (p < 0.05). Additionally, the prevalence of arthritis was higher among women with endometriosis (25.93%) compared to those without endometriosis (13.64%). Notably, no significant differences were observed between the two groups concerning BMI, education level, smoking, alcohol status, PA, CKD, DM, hyperlipidemia, and hypertension.

**Table 1:** Characteristics of women aged 20-54 years from NHANES 1999-2006, weighted.

Variable	Total	Endometriosis		*P value*
		No	Yes	
**Overall, n**	2915	2672	243	
Age, years, mean ± SD	38.20 (0.25)	37.89 (0.27)	41.11 (0.49)	***< 0.0001***
BMI, kg/m^2^, mean ± SD	27.87 (0.22)	27.88 (0.23)	27.73 (0.51)	0.78
Race, n (%)				***< 0.001***
Non-Hispanic White	1568 (74.83)	1395 (73.66)	173 (85.60)	
Non-Hispanic Black	552 (9.63)	515 (9.96)	37 (6.57)	
Mexican American	565 (6.33)	545 (6.75)	20 (2.38)	
Other Hispanic	127 (5.16)	122 (5.51)	5 (1.95)	
Other Race	103 (4.06)	95 (4.12)	8 (3.50)	
Marital status, n (%)				***0.01***
Married/Living with partner	1831 (66.30)	1669 (65.85)	162 (70.46)	
Never married	598 (17.75)	571 (18.64)	27 (9.49)	
Divorced/Separated/Widowed	486 (15.96)	432 (15.51)	54 (20.05)	
Education level, n (%)				0.06
College graduate or above	740 (29.74)	679 (30.16)	61 (25.83)	
Some college or AA degree	1054 (37.12)	967 (37.52)	87 (33.35)	
High school/GED/Less than 11th grade	1121 (33.14)	1026 (32.31)	95 (40.82)	
Smoking, n (%)				0.27
No	1748 (57.34)	1615 (57.76)	133 (53.42)	
Yes	1167 (42.66)	1057 (42.24)	110 (46.58)	
Alcohol status, n (%)				0.05
Never	404 (11.83)	381 (12.02)	23 (10.12)	
Former	401 (12.65)	358 (12.15)	43 (17.24)	
Mild/Moderate	1470 (53.45)	1338 (53.19)	132 (55.83)	
Heavy	640 (22.08)	595 (22.65)	45 (16.81)	
Income to poverty ratio, mean ± SD	3.16 (0.05)	3.13 (0.06)	3.42 (0.12)	***0.03***
Physical activity, MET-h/week, mean ± SD	847.06 (38.05)	844.79 (40.18)	868.01 (84.98)	0.80
SUA, μmol/L, mean ± SD	266.49 (1.65)	265.51 (1.60)	275.56 (4.27)	***0.01***
Chronic kidney disease, n (%)				0.67
No	2664 (92.61)	2441 (92.52)	223 (93.47)	
Yes	251 (7.39)	231 (7.48)	20 (6.53)	
Diabetes mellitus, n (%)				0.44
No	2774 (96.18)	2540 (96.08)	234 (97.10)	
Yes	141 (3.82)	132 (3.92)	9 (2.90)	
Hyperlipidemia, n (%)				0.30
No	1017 (35.38)	936 (35.71)	81 (32.33)	
Yes	1898 (64.62)	1736 (64.29)	162 (67.67)	
Hypertension, n (%)				0.08
No	2270 (78.57)	2104 (79.11)	166 (73.60)	
Yes	645 (21.43)	568 (20.89)	77 (26.40)	
Arthritis, n (%)				***< 0.0001***
No	2496 (85.16)	2319 (86.36)	177 (74.07)	
Yes	419 (14.84)	353 (13.64)	66 (25.93)	

*All estimates accounted for sample weights and complex survey designs, and percentages were adjusted for survey weights of NHANES. BMI, body mass index; GED, general educational development; NHANES, National Health and Nutrition Examination Survey; SD, standard deviation; SUA, serum uric acid; *p* value in bold indicates statistical significance.

### 3.2 Associations between endometriosis and arthritis from NHANES

The relationships between endometriosis and the risk of arthritis were investigated through multivariate regression models, and the results are presented in **Table 2**. In crude model, the odds ratio (OR) for endometriosis and arthritis was 2.22 (95% CI: 1.61, 3.05; *p < 0.0001*). These findings remained consistent across different adjustment models, namely model 1 (OR: 2.01; 95% CI: 1.42, 2.85; *p < 0.001*), model 2 (OR: 1.87; 95% CI: 1.32, 2.66; *p < 0.001*), and model 3 (OR: 1.89; 95% CI: 1.33, 2.67; *p < 0.001*). Additionally, we explored the associations between endometriosis and different types of arthritis, showed in **Table 3**. The analysis revealed a positive significant association between endometriosis and RA, with an OR of 2.07 (95% CI: 1.03, 4.17; *p = 0.04*). The baseline data of patients with RA were shown in **Supplementary Table 3**. Similarly, endometriosis also showed positive significant associations with other types of arthritis (OR: 2.78; 95% CI: 1.30, 5.95; *p = 0.01*), and cases that the arthritis type was unknown (OR: 2.06; 95% CI: 1.36, 3.11; *p = 0.001*). However, the associations between endometriosis and osteoarthritis were not statistically significant, with an OR of 1.40 (95% CI: 0.70, 2.80; p = 0.33).

**Table 2:** Associations between endometriosis and the risk of arthritis in U.S. women from NHANES 1999-2006.

	Total, n(%)	Arthritis, n(%)	Crude model		Model 1		Model 2		Model 3	
			95% CI	*P value*	95% CI	*P value*	95% CI	*P value*	95% CI	*P value*
Endometriosis										
**No**	2672 (90.25)	353 (82.96)	ref		ref		ref		ref	
**Yes**	243 (9.75)	66 (17.04)	2.22 (1.61, 3.05)	***< 0.0001***	2.01 (1.42, 2.85)	***< 0.001***	1.87 (1.32, 2.66)	***< 0.001***	1.89 (1.33, 2.67)	***< 0.001***

Crude model was unadjusted. Model 1 was adjusted for Age (continuous) + BMI (continuous). Model 2 was adjusted for model 1 + Race (White, Black, Mexican, Hispanic, Other race), Marital status (Married/Living with partner, Never married, Divorced/Separated/Widowed), Education level (College graduate or above, Some college or AA degree, High school/GED/Less than 11th grade), Smoking status (No, Yes), Alcohol status (Never, Former, Mild/Moderate, Heavy), PIR (continuous), and Physical activity (continuous). Model 3 was adjusted for model 2 + serum uric acid (continuous), Chronic kidney disease (No, Yes) + Diabetes mellitus (No, Yes) + Hyperlipidemia (No, Yes) + Hypertension (No, Yes). CI, confidence interval; NHANES, National Health and Nutrition Examination Survey; OR, odds ratios; Ref, reference group. *p* value in bold indicates statistical significance.

**Table 3:** Associations between endometriosis and the risk of different arthritis in U.S. women from NHANES 1999-2006.

	Total, n(%)	Case, n(%)	Crude model		Model 1		Model 2		Model 3	
			95% CI	*P value*	95% CI	*P value*	95% CI	*P value*	95% CI	*P value*
Rheumatoid arthritis										
** Endometriosis**										
** No**	2399 (91.23)	80 (82.44)	Ref		ref		ref		ref	
** Yes**	192 (8.77)	15 (17.56)	2.30 (1.23, 4.30)	***0.01***	2.10 (1.11, 3.96)	***0.02***	2.09 (1.06, 4.10)	***0.03***	2.07 (1.03, 4.17)	***0.04***
Osteoarthritis										
** Endometriosis**										
** No**	2412 (91.23)	93 (85.89)	Ref		ref		ref		ref	
** Yes**	192 (8.77)	15 (14.11)	1.77 (0.98, 3.20)	0.06	1.58 (0.86, 2.89)	0.14	1.42 (0.74, 2.70)	0.28	1.40 (0.70, 2.80)	0.33
Other										
** Endometriosis**										
** No**	2378 (91.19)	59 (79.25)	Ref		ref		ref		ref	
** Yes**	189 (8.81)	12 (20.75)	2.83 (1.36, 5.87)	***0.01***	2.57 (1.19, 5.58)	***0.02***	2.84 (1.33, 6.09)	***0.01***	2.78 (1.30, 5.95)	***0.01***
Do not know arthritis type										
** Endometriosis**										
** No**	2440 (91.00)	121 (82.36)	Ref		ref		ref		ref	
** Yes**	201 (9.00)	24 (17.64)	2.31 (1.56, 3.43)	***< 0.0001***	2.08 (1.36, 3.18)	***0.001***	2.02(1.31, 3.11)	***0.002***	2.06 (1.36, 3.11)	***0.001***

Crude model was unadjusted. Model 1 was adjusted for Age (continuous) + BMI (continuous). Model 2 was adjusted for model 1 + Race (White, Black, Mexican, Hispanic, Other race), Marital status (Married/Living with partner, Never married, Divorced/Separated/Widowed), Education level (College graduate or above, Some college or AA degree, High school/GED/Less than 11th grade), Smoking status (No, Yes), Alcohol status (Never, Former, Mild/Moderate, Heavy), PIR (continuous), and Physical activity (continuous). Model 3 was adjusted for model 2 + serum uric acid (continuous), Chronic kidney disease (No, Yes) + Diabetes mellitus (No, Yes) + Hyperlipidemia (No, Yes) + Hypertension (No, Yes). CI, confidence interval; NHANES, National Health and Nutrition Examination Survey; OR, odds ratios; Ref, reference group. *p* value in bold indicates statistical significance.

### 3.3 Subgroup and interactive analyses of endometriosis and arthritis from NHANES

**Figure 2:**
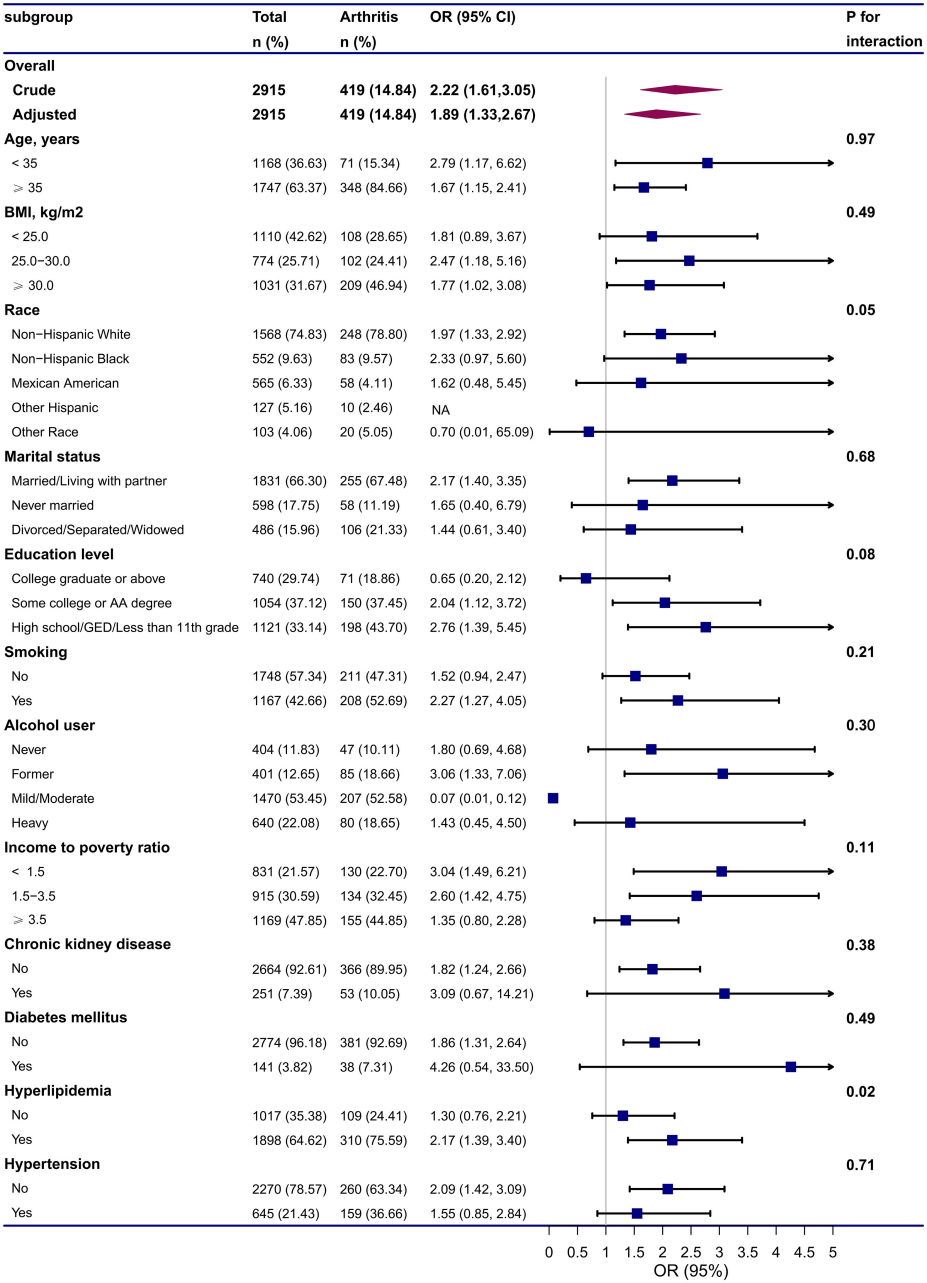
Subgroup and interactive analyses of endometriosis and arthritis from NHANES. Adjusted for Age (continuous), BMI (continuous), Race (White, Black, Mexican, Hispanic, Other race), Marital status (Married/Living with partner, Never married, Divorced/Separated/Widowed), Education level (College graduate or above, Some college or AA degree, High school/GED/Less than 11th grade), Smoking status (No, Yes), Alcohol status (Never, Former, Mild/Moderate, Heavy), PIR (continuous), Physical activity (continuous), serum uric acid (continuous), Chronic kidney disease (No, Yes), Diabetes mellitus (No, Yes), Hyperlipidemia (No, Yes), Hypertension (No, Yes). All covariates in the subgroup analysis models were adjusted, except the stratification variable itself (for example, “age” was not included as a covariate in the age subgroup). CI, confidence interval; GED, general educational development; OR, odds ratios; NA, not applicable.

We performed subgroup analyses to assess the stability of the association between endometriosis and arthritis in different populations based on age, BMI, race, marital status, education level, smoking status, alcohol intake, PIR, CKD, DM, hyperlipidemia, and hypertension (**Figure 2**). All covariates in each subgroup analysis model were adjusted, except the stratification variable itself. The results showed that endometriosis was positively associated with arthritis in different subgroups, except subgroups of other race, college graduate or above, and mild/moderate alcohol user. No significant interactions were observed, indicated that the positive correlation between endometriosis and arthritis was also not affected by the interaction among different subgroups (all *p* for interaction > 0.05), except subgroup of hyperlipidemia (*p* for interaction = 0.02).

### 3.4 Causal relationship between endometriosis and RA from genetic correlation analysis and MR analysis

As the significantly positive correlation between endometriosis and RA was observed using NHANES 1999-2006 database, we further conducted genetic correlation analysis and MR analysis to deduce the causal relationship between endometriosis on RA. We did not find an overall genetic correlation between endometriosis and sets of RA (all P value >0.05), as shown in **Table 4**. This result suggests endometriosis and RA do not share a genetic relationship, or that their potential relationship is not related to genetics.

**Table 4:** Genetic correlation analysis between endometriosis and RA.

Trait1	Trait2	r_g_	r_g__SE	P value
Endometriosis	Rheumatoid arthritis (ukb-b-11874)	0.096	0.179	0.589
	Rheumatoid arthritis (finn-b-M13_RHEUMA)	0.256	0.237	0.282
	Other rheumatoid arthritis (ukb-b-M06)	0.251	0.270	0.353

**Figure 3:**
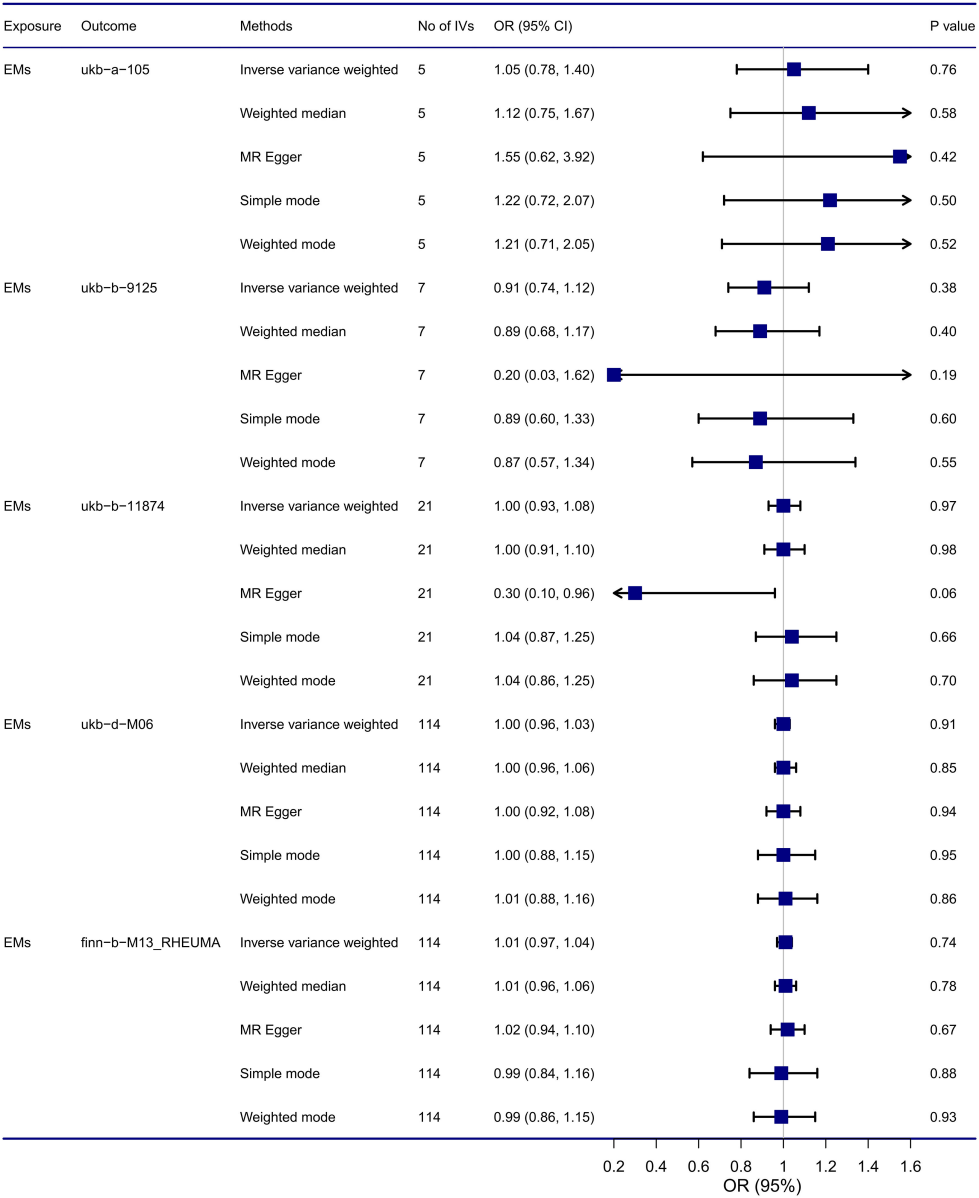
Causal relationship between endometriosis and RA from MR analysis. CI, confidence interval; EMs, endometriosis; IVs, instrumental variables; OR, odds ratios.

Results from MR analysis (**Figure 3**) suggested that no causal relationship was found between endometriosis and RA using the IVW method, the OR of five GWAS for RA were 1.05 (95% CI: 0.78, 1.40), 0.91 (95% CI: 0.74, 1.12), 1.00 (95% CI: 0.93, 1.08), 1.00 (95% CI: 0.96, 1.03), and 1.01 (95% CI: 0.97, 1.04), respectively. Additionally, these results were consistent with other supplementary MR methods (Weighted median, MR Egger, Simple mode, Weighted mode) in terms of the causal relationship estimation, thus indicating that there was no causal relationship between endometriosis and RA, and these findings are reliable and robust. Scatter plots for MR also revealed that no causal relationship was found between endometriosis and RA, shown in **Supplementary Figure 1**.

As a result of the sensitivity analysis, no horizontal pleiotropy (all P intercept > 0.05, shown in **Supplementary Table 4**), heterogeneity (all P value for Cochran’s Q test > 0.05, and the funnel plot presents a symmetric property, shown in **Supplementary Table 5** and **Supplementary Figure 2**), and reverse causality (all correct causal direction were true, and all P value for MR-Steiger < 0.05, shown in **Supplementary Table 6**) were found in the selected instruments. These results were confirmed by leave−one−out sensitivity analysis (**Supplementary Figure 3**). Thus, the sensitivity analysis supports the robustness of the main findings, indicating no causal relationship between endometriosis and RA.

## 4 Discussion

In this study, we integrated a cross-sectional study using NHANES 1999-2006, genetic correlation analysis, and MR analysis using GWAS summary statistics to investigate the association between endometriosis and arthritis. Our finding suggested that endometriosis had positive association with arthritis, especially with RA, after controlling for potential confounders of age, BMI, race, marital status, education level, smoking status, alcohol status, PIR, PA, SUA, CKD, DM, hyperlipidemia, hypertension. However, the results of genetic correlation analysis and MR analysis did not support a causal association between endometriosis and RA, suggesting that their potential relationship is not related to genetics.

Endometriosis is a chronic systemic, inflammatory gynecologic disorder, associated with a wide range of symptoms and high-risk comorbidities (1). Accumulating evidence implicates a positive correlation between endometriosis and autoimmune diseases, including systemic lupus erythematosus, Sjogren’s syndrome, ankylosing spondylitis, psoriatic, rheumatoid arthritis (8, 11, 14, 17, 34, 35). The Nurses’ Health Study II (N = 114,453, over a 22-year period of follow-up) suggested an association between endometriosis and risk of RA (HR = 1.41; 95% CI: 1.05, 1.89) (11). Similarly, another large nationwide population-based cohort study(N = 28, 926, over a 13-year period of follow-up) from Taiwan reported patients with endometriosis had a higher risk of RA (HR = 1.75; 95% CI: 1.27, 2.41) (16). Moreover, a large-scale nested case-control study (30, 516 cases with endometriosis and 120, 976 control participants) from Japan released that there were significant positive associations between endometriosis and RA, the incidence rate ratio was 1.31 (95% CI: 1.05, 1.64) (17). In addition, a meta-analysis from two cross-sectional studies showed that compared to the general female population, women with endometriosis had a significantly greater risk of RA (OR = 1.50,95% CI: 1.18,1.91) (8). Recently, another meta-analysis findings indicated that endometriosis increases the risk of RA, with a RR of 1.89 (95% CI: 1.04, 3.42) in cohort studies and an OR of 1.40 (95% CI: 1.19, 1.64) in case-control and cross-sectional studies (15). Consistently to those studies, in our cross-sectional study, we also found endometriosis had positive association with arthritis (OR = 1.89; 95% CI: 1.33, 2.67). Particularly noteworthy is the heightened association between endometriosis and RA, with an OR of 2.07 (95% CI: 1.03, 4.17) after adjusting for multiple variables.

The limitations of cross-sectional studies restrict their ability to establish a causal relationship between endometriosis and RA. Challenges arise in discerning the sequence of disease development and manifestation, as well as identifying potential causal relationships. In cohort studies, unknown confounding factors may influence the results, and we cannot exclude the possibility that unmeasured confounding factors affect the reported associations between endometriosis and RA. Therefore, based on the significantly positive correlation between endometriosis and RA was observed from a cross-sectional study using NHANES 1999-2006 database, we further performed the genetic correlation analysis and MR analysis to clarify the causal relationship between endometriosis and RA. LDSC regression, a widely employed method for genetic correlation analysis, enables the assessment of single nucleotide variant-based phenotype heritability and coheritability between two traits (19, 29). MR analysis, less susceptible to biases from confounding factors and reverse causality (36), can be utilized to explore the causal relationship between endometriosis and RA.

The findings from gene association studies and/or GWASs have indicated the presence of genetic polymorphisms between endometriosis and RA, suggesting a shared a similar genetic background between these two conditions (18). Some of the implicated genes include protein tyrosine phosphatase non-receptor type 22 gene (37), chemokine CC motif ligand 21 gene, human leukocytes antigen (HLA)-DRB1 gene (37, 38), and IL-6 gene (39, 40). However, a comprehensive genetic correlation analysis of endometriosis and its comorbidity with other conditions found that none of the autoimmune conditions, such as celiac disease, Crohn’s disease, inflammatory bowel disease, ulcerative colitis, systemic lupus erythematosus, RA, and multiple sclerosis, demonstrated a significant genetic correlation with endometriosis (27). Similarly, based on GWAS meta-analysis summary statistical data, LDSC regression analysis between endometriosis and RA did not reveal any significant findings in our study (all P values > 0.05). Moreover, to ascertain the causal role of endometriosis in RA, MR analysis was undertaken using five GWAS summary datasets from the European population. The MR results did not provide evidence supporting a causal relationship between endometriosis and RA, indicating that their potential relationship is not genetically driven. These results remained consistent across various MR methods, including the primary IVW method, and supplementary MR approaches corroborated the reliability of this non-causal relationship. However, evidence from a recent MR study by Tang T et al. found that the results of the IVW model suggested a causal association between genetic predisposition to endometriosis and an increased risk of RA (OR = 1.005, 95% CI: 1.001, 1.009, p = 0.014). Considering that this study only used GWAS summary data for both the exposure (endometriosis) and the outcome (RA), their results need to be interpreted with caution. In our study, we utilized a GWAS meta-analysis to integrate data from three GWAS studies on endometriosis (as shown in **Supplementary Table 1**), thereby increasing the sample size, improving statistical power, and more accurately identifying genetic factors associated with the diseases or phenotypes (28). Additionally, the summary statistics on RA used in our study were obtained from five GWAS (as shown in **Supplementary Table 1**), which significantly strengthened the evidence supporting our conclusions.

Observational studies have noted a positive association between endometriosis and RA, yet genetic correlation analysis and MR analysis suggest that this relationship is not causal. Rather than being driven by intrinsic genetic inheritance, it appears that other extrinsic factors may underlie this association. Several studies have proposed that the positive association between endometriosis and RA could be attributed to the imbalance of the immune system in clinical practice (8). Endometriosis, characterized as a systemic inflammatory disease, may serve as a driver of RA. Alterations in cell-mediated and humoral immunity observed in patients with endometriosis could potentially explain the heightened risk of RA in this population (8). Numerous studies have shown increased concentrations of interleukins (IL-1, IL-6, IL-8, and IL-33), tumor necrosis factor-alpha, insulin-like growth factor 1, and vascular endothelial growth factor in endometriosis patients, among endometriosis lesions and peritoneal fluid (4, 41–44). Additionally, it’s been observed that patients with endometriosis often exhibit an increase in neutrophils and macrophages within their peritoneal fluid (45). The meta-analysis findings indicate that there is an elevation in B lymphocytes and excessive production of autoantibodies in endometriosis (46). Elevated proinflammatory cytokines and alterations in immune cell populations contribute to the creation of a broad inflammatory environment in endometriosis, extending beyond the pelvic region. These factors may also play a crucial role in mediating the pathogenesis of RA. Finally, it’s worth noting that endometriosis and RA primarily occurs in women of reproductive age. This is because hormonal factors also play a significant role in the development of both conditions (4, 47).

The advantage of our study lies in its comprehensive approach to investigating the causal relationship between endometriosis and arthritis. We employed three study design methods: observational study, genetic correlation analysis, and MR study, enabling a thorough exploration of this relationship. The multivariable regression models and subgroup analyses among different subgroups provide the stability of the positive relationship between endometriosis and RA. Furthermore, the genetic correlation analysis and MR analysis can avoid unmeasured confounding and reverse causality bias.

However, our study also has several limitations. Firstly, participants’ self-reported diagnoses of endometriosis and arthritis were based on whether a doctor or other health professional had ever told them they had the condition, which can reduce bias caused by participants. However, this approach may still inevitably introduce recall bias in this study. Secondly, in the NHANES 1999-2006 cycle, the categorization of arthritis types included rheumatoid arthritis, osteoarthritis, “don’t know”, and “other” category without explicit definition of the specific types included. This lack of detailed categorization may limit the precision of our findings regarding arthritis subtypes. Third, our study primarily focused on European and American populations, which may restrict the generalizability of our findings to other ethnic groups. This limitation underscores the need for future research with larger and more diverse sample sizes to validate our findings across different ethnic populations. Additionally, our study does not directly explain the mechanism underlying the relationship between endometriosis and RA, which will require future investigation through specific cell and animal experiments. We will further study this issue in future research. Finally, although multiple variables were adjusted for, there may still be unmeasured confounding factors that could influence the results, which is an unavoidable deficiency of observational studies. To address this, we employed LDSC and MR analyses, which use genetic variation as a natural experiment to explore genetic correlations and infer causality from observational data, thereby reducing confounding from unmeasured variables. However, LDSC and MR analyses suggested that their potential relationship is not related to genetics. Therefore, well-designed large prospective cohort studies with more confounding control and standardized data collections are needed in the future to further validate these results.

## 5 Conclusions

In conclusion, a cross-sectional study identified a significant positive association between endometriosis and arthritis among US women aged 20-54 years from NHANES 1999-2006, particularly with RA. However, findings based on genetic correlation analysis and MR analysis did not support a genetic correlation or causal role. These findings suggest that clinicians should pay more attention to the coexistence of RA in endometriosis patients and explore the shared pathophysiological mechanisms of these two disorders, with a particular focus on extrinsic factors rather than intrinsic genetic inheritance.

## Data availability statement

Publicly available datasets were analyzed in this study. This data can be found here: The dataset can be downloaded free of charge from the NHANES official website (https://www.cdc.gov/nchs/nhanes/index.htm) (accessed on 28 Apr 2024). The GWAS studies in this study are included in **Supplementary Table 1**. Further inquiries can be directed to the corresponding author.

## Ethics statement

The studies involving humans were approved by the National Center for Health Statistics Institutional Review Board, the North West Multi-Centre Research Ethics Committee (REC reference: 16/NW/0274), the Coordinating Ethics Committee of the Helsinki and Uusimaa Hospital District (Nr HUS/990/2017). The studies were conducted in accordance with the local legislation and institutional requirements. The participants provided their written informed consent to participate in this study.

## Author contributions

HX: Writing – review & editing, Writing – original draft, Methodology, Investigation, Formal analysis, Data curation, Conceptualization. HZ: Writing – original draft, Visualization, Formal analysis. QW: Writing – original draft, Visualization, Formal analysis. XX: Writing – review & editing, Investigation, Data curation. NX: Writing – review & editing, Supervision, Investigation. SW: Writing – review & editing, Methodology, Conceptualization.

## References

[1] Horne AW Missmer SA. Pathophysiology, diagnosis, and management of endometriosis. BMJ. (2022) 379:e070750. doi: 10.1136/bmj-2022-070750 36375827

[2] Zondervan KT Becker CM Missmer SA. Endometriosis. N Engl J Med. (2020) 382:1244–56. doi: 10.1056/NEJMra1810764 32212520

[3] Taylor HS Kotlyar AM Flores VA. Endometriosis is a chronic systemic disease: clinical challenges and novel innovations. Lancet. (2021) 397:839–52. doi: 10.1016/S0140-6736(21)00389-5 33640070

[4] Saunders PTK Horne AW. Endometriosis: etiology, pathobiology, and therapeutic prospects. Cell. (2021) 184:2807–24. doi: 10.1016/j.cell.2021.04.041 34048704

[5] Thiel PS Bougie O Pudwell J Shellenberger J Velez MP Murji A. Endometriosis and mental health: A population-based cohort study. Am J Obstet Gynecol. (2024) 230(6):649.e1–.e19. doi: 10.1016/j.ajog.2024.01.023 38307469

[6] Wei Y Liang Y Lin H Dai Y Yao S. Autonomic nervous system and inflammation interaction in endometriosis-associated pain. J Neuroinflamm. (2020) 17:80. doi: 10.1186/s12974-020-01752-1 PMC7060607 32145751

[7] Clower L Fleshman T Geldenhuys WJ Santanam N. Targeting oxidative stress involved in endometriosis and its pain. Biomolecules. (2022) 12(8):1055. doi: 10.3390/biom12081055 36008949 PMC9405905

[8] Shigesi N Kvaskoff M Kirtley S Feng Q Fang H Knight JC. The association between endometriosis and autoimmune diseases: a systematic review and meta-analysis. Hum Reprod Update. (2019) 25:486–503. doi: 10.1093/humupd/dmz014 31260048 PMC6601386

[9] Pasquini B Seravalli V Vannuccini S La Torre F Geppetti P Iannone L. Endometriosis and the diagnosis of different forms of migraine: an association with dysmenorrhoea. Reprod BioMed Online. (2023) 47:71–6. doi: 10.1016/j.rbmo.2023.03.020 37202318

[10] McGrath IM Montgomery GW Mortlock S. Insights from mendelian randomization and genetic correlation analyses into the relationship between endometriosis and its comorbidities. Hum Reprod Update. (2023) 29:655–74. doi: 10.1093/humupd/dmad009 PMC10477944 37159502

[11] Harris HR Costenbader KH Mu F Kvaskoff M Malspeis S Karlson EW. Endometriosis and the risks of systemic lupus erythematosus and rheumatoid arthritis in the nurses' Health study ii. Ann Rheum Dis. (2016) 75:1279–84. doi: 10.1136/annrheumdis-2015-207704 PMC4740245 26238146

[12] Blom JN Velez MP McClintock C Shellenberger J Pudwell J Brogly SB. Endometriosis and cardiovascular disease: a population-based cohort study. CMAJ Open. (2023) 11:E227–E36. doi: 10.9778/cmajo.20220144 PMC10000901 36882211

[13] Kvaskoff M Mahamat-Saleh Y Farland LV Shigesi N Terry KL Harris HR. Endometriosis and cancer: a systematic review and meta-analysis. Hum Reprod Update. (2021) 27:393–420. doi: 10.1093/humupd/dmaa045 33202017

[14] Harris HR Korkes KMN Li T Kvaskoff M Cho E Carvalho LF. Endometriosis, psoriasis, and psoriatic arthritis: a prospective cohort study. Am J Epidemiol. (2022) 191:1050–60. doi: 10.1093/aje/kwac009 PMC9393059 35029650

[15] Tang T Zhong Y Xu S Yu H. Causal effects of endometriosis on sle, ra and ss risk: evidence from meta-analysis and mendelian randomization. BMC Pregnancy Childbirth. (2024) 24:162. doi: 10.1186/s12884-024-06347-9 38395801 PMC10885476

[16] Xue YH You LT Ting HF Chen YW Sheng ZY Xie YD. Increased risk of rheumatoid arthritis among patients with endometriosis: A nationwide population-based cohort study. Rheumatol (Oxford). (2021) 60:3326–33. doi: 10.1093/rheumatology/keaa784 33331948

[17] Yoshii E Yamana H Ono S Matsui H Yasunaga H. Association between allergic or autoimmune diseases and incidence of endometriosis: a nested case-control study using a health insurance claims database. Am J Reprod Immunol. (2021) 86:e13486. doi: 10.1111/aji.13486 34322942

[18] Zervou MI Vlachakis D Papageorgiou L Eliopoulos E Goulielmos GN. Increased risk of rheumatoid arthritis in patients with endometriosis: genetic aspects. Rheumatol (Oxford). (2022) 61:4252–62. doi: 10.1093/rheumatology/keac143 35258592

[19] Bulik-Sullivan BK Loh PR Finucane HK Ripke S Yang J Schizophrenia Working Group of the Psychiatric Genomics C. Ld score regression distinguishes confounding from polygenicity in genome-wide association studies. Nat Genet. (2015) 47:291–5. doi: 10.1038/ng.3211 PMC4495769 25642630

[20] Larsson SC Butterworth AS Burgess S. Mendelian randomization for cardiovascular diseases: principles and applications. Eur Heart J. (2023) 44:4913–24. doi: 10.1093/eurheartj/ehad736 PMC10719501 37935836

[21] Li J Li C Huang Y Guan P Huang D Yu H. Mendelian randomization analyses in ocular disease: a powerful approach to causal inference with human genetic data. J Trans Med. (2022) 20(1):621. doi: 10.1186/s12967-022-03822-9 PMC9793675 36572895

[22] Sun L Ye Z Ling Y Cai S Xu J Fan C. Relationship between polycyclic aromatic hydrocarbons and rheumatoid arthritis in us general population, nhanes 2003-2012. Sci Total Environ. (2020) 704:135294. doi: 10.1016/j.scitotenv.2019.135294 31791769

[23] Liu B Wang J Li YY Li KP Zhang Q. The association between systemic immune-inflammation index and rheumatoid arthritis: evidence from nhanes 1999-2018. Arthritis Res Ther. (2023) 25:34. doi: 10.1186/s13075-023-03018-6 36871051 PMC9985219

[24] Lei T Qian H Yang J Hu Y. The exposure to volatile organic chemicals associates positively with rheumatoid arthritis: A cross-sectional study from the nhanes program. Front Immunol. (2023) 14:1098683. doi: 10.3389/fimmu.2023.1098683 37404817 PMC10317299

[25] Vilar-Gomez E Nephew LD Vuppalanchi R Gawrieh S Mladenovic A Pike F. High-quality diet, physical activity, and college education are associated with low risk of nafld among the us population. Hepatology. (2022) 75:1491–506. doi: 10.1002/hep.32207 34668597

[26] Kidney Disease: Improving Global Outcomes Glomerular Diseases Work G. Kdigo. Clinical practice guideline for the management of glomerular diseases. Kidney Int. (2021) 100:S1–S276. doi: 10.1016/j.kint.2021.05.021 34556256

[27] Rahmioglu N Mortlock S Ghiasi M Moller PL Stefansdottir L Galarneau G. The genetic basis of endometriosis and comorbidity with other pain and inflammatory conditions. Nat Genet. (2023) 55:423–36. doi: 10.1038/s41588-023-01323-z PMC10042257 36914876

[28] Defo J Awany D Ramesar R. From snp to pathway-based gwas meta-analysis: do current meta-analysis approaches resolve power and replication in genetic association studies? Brief Bioinform. (2023) 24(1):bbac600. doi: 10.1093/bib/bbac600 36611240

[29] Bulik-Sullivan B Finucane HK Anttila V Gusev A Day FR Loh PR. An atlas of genetic correlations across human diseases and traits. Nat Genet. (2015) 47:1236–41. doi: 10.1038/ng.3406 PMC4797329 26414676

[30] Skrivankova VW Richmond RC Woolf BAR Yarmolinsky J Davies NM Swanson SA. Strengthening the reporting of observational studies in epidemiology using mendelian randomization: the strobe-mr statement. JAMA. (2021) 326:1614–21. doi: 10.1001/jama.2021.18236 34698778

[31] Liu D Cao M Wang H Cao W Zheng C Li Y. Association between inflammatory bowel disease and cancer risk: evidence triangulation from genetic correlation, mendelian randomization, and colocalization analyses across east asian and european populations. BMC Med. (2024) 22(1):137. doi: 10.1186/s12916-024-03352-9 38528540 PMC10964701

[32] Boehm FJ Zhou X. Statistical methods for mendelian randomization in genome-wide association studies: a review. Comput Struct Biotechnol J. (2022) 20:2338–51. doi: 10.1016/j.csbj.2022.05.015 PMC9123217 35615025

[33] Mavromatis LA Rosoff DB Cupertino RB Garavan H Mackey S Lohoff FW. Association between brain structure and alcohol use behaviors in adults: a mendelian randomization and multiomics study. JAMA Psychiatry. (2022) 79:869–78. doi: 10.1001/jamapsychiatry.2022.2196 PMC9366661 35947372

[34] Yin Z Low HY Chen BS Huang KS Zhang Y Wang YH. Risk of ankylosing spondylitis in patients with endometriosis: a population-based retrospective cohort study. Front Immunol. (2022) 13:877942. doi: 10.3389/fimmu.2022.877942 35784295 PMC9240188

[35] Chao YH Liu CH Pan YA Yen FS Chiou JY Wei JC. Association between endometriosis and subsequent risk of sjogren's syndrome: a nationwide population-based cohort study. Front Immunol. (2022) 13:845944. doi: 10.3389/fimmu.2022.845944 35592328 PMC9110644

[36] Davies NM Holmes MV Davey Smith G. Reading mendelian randomisation studies: a guide, glossary, and checklist for clinicians. BMJ. (2018) 362:k601. doi: 10.1136/bmj.k601 30002074 PMC6041728

[37] Li PF Li S Zheng PS. Reproductive effect by rheumatoid arthritis and related autoantibodies. Rheumatol Ther. (2024) 11:239–56. doi: 10.1007/s40744-023-00634-1 PMC10920578 38376734

[38] Sundqvist J Falconer H Seddighzadeh M Vodolazkaia A Fassbender A Kyama C. Endometriosis and autoimmune disease: association of susceptibility to moderate/severe endometriosis with ccl21 and hla-drb1. Fertil Steril. (2011) 95:437–40. doi: 10.1016/j.fertnstert.2010.07.1060 20797713

[39] Shao M Xie H Yang H Xu W Chen Y Gao X. Association of interleukin-6 promoter polymorphism with rheumatoid arthritis: A meta-analysis with trial sequential analysis. Clin Rheumatol. (2022) 41:411–9. doi: 10.1007/s10067-021-05886-2 34494214

[40] Wang XQ Hu M Chen JM Sun W Zhu MB. Effects of gene polymorphism and serum levels of il-2 and il-6 on endometriosis. Eur Rev Med Pharmacol Sci. (2020) 24:4635–41. doi: 10.26355/eurrev_202005_21148 32432788

[41] Forster R Sarginson A Velichkova A Hogg C Dorning A Horne AW. Macrophage-derived insulin-like growth factor-1 is a key neurotrophic and nerve-sensitizing factor in pain associated with endometriosis. FASEB J. (2019) 33:11210–22. doi: 10.1096/fj.201900797R PMC6766660 31291762

[42] Kato T Yasuda K Matsushita K Ishii KJ Hirota S Yoshimoto T. Interleukin-1/-33 signaling pathways as therapeutic targets for endometriosis. Front Immunol. (2019) 10:2021. doi: 10.3389/fimmu.2019.02021 31507610 PMC6714064

[43] Banerjee S Xu W Doctor A Driss A Nezhat C Sidell N. Tnfalpha-induced altered mirna expression links to nf-kappab signaling pathway in endometriosis. Inflammation. (2023) 46:2055–70. doi: 10.1007/s10753-023-01862-x PMC10673760 37389684

[44] Hung SW Zhang R Tan Z Chung JPW Zhang T Wang CC. Pharmaceuticals targeting signaling pathways of endometriosis as potential new medical treatment: A review. Med Res Rev. (2021) 41:2489–564. doi: 10.1002/med.21802 PMC8252000 33948974

[45] Hogg C Horne AW Greaves E. Endometriosis-associated macrophages: origin, phenotype, and function. Front Endocrinol (Lausanne). (2020) 11:7. doi: 10.3389/fendo.2020.00007 32038499 PMC6989423

[46] Riccio LGC Baracat EC Chapron C Batteux F Abrao MS. The role of the B lymphocytes in endometriosis: A systematic review. J Reprod Immunol. (2017) 123:29–34. doi: 10.1016/j.jri.2017.09.001 28910679

[47] Cutolo M Straub RH. Sex steroids and autoimmune rheumatic diseases: state of the art. Nat Rev Rheumatol. (2020) 16:628–44. doi: 10.1038/s41584-020-0503-4 33009519

