# Supplementary material for: Association between endometriosis and arthritis: results from NHANES 1999-2006, genetic correlation analysis, and Mendelian randomization study
Source: Front Immunol. 2024 Jul 29;15:1424648. doi: 10.3389/fimmu.2024.1424648 (PMC11317389; doi:10.3389/fimmu.2024.1424648)
Supplement: Supplementary file 1 [file Table_1.docx]

Supplementary Table 1. Details of GWAS studies included in our analysis

| Trait | No. of case subjects | No. of control subjects | Consortium or GWAS ID | Population |
| --- | --- | --- | --- | --- |
| Endometriosis | | | | |
|  | 16,588 | 111,583 | FinnGen consortium release data (finn-b-N14_ENDOMETRIOSIS), data description: [r9.finngen.fi/pheno/N14_ENDOMETRIOSIS](https://r9.finngen.fi/pheno/N14_ENDOMETRIOSIS), data download from https://storage.googleapis.com/finngen-public-data-r9/summary_stats/finngen_R9_N14_ENDOMETRIOSIS.gz | European |
|  | 1,496 | 359,698 | [UK BIOBANK](http://www.ukbiobank.ac.uk/) release data(ukb-b-N80) , download from https://gwas.mrcieu.ac.uk/datasets/ukb-d-N80/) | European |
|  | 1,937 | 245,603 | Jiang L et al. (PMID: [34737426](https://www.ebi.ac.uk/gwas/publications/34737426), download from https://www.ebi.ac.uk/gwas/home | European |
| Rheumatoid arthritis | | | | |
|  | 3,730 | 333,429 | ukb-a-105, download from https://gwas.mrcieu.ac.uk/datasets/ukb-b-105/ | European |
|  | 5,201 | 457,732 | ukb-b-9125, download from https://gwas.mrcieu.ac.uk/datasets/ukb-b-9125/ | European |
|  | 1,523 | 461,487 | ukb-b-11874, download from https://gwas.mrcieu.ac.uk/datasets/ukb-b-11874/ | European |
|  | 1,401 | 359,793 | ukb-d-M06, download from https://gwas.mrcieu.ac.uk/datasets/ukb-d-M06/ | European |
|  | 12,555 | 262,844 | FinnGen consortium release data (finn-b-M13_RHEUMA), data description: [r9.finngen.fi/pheno/M13_RHEUMA](https://r9.finngen.fi/pheno/M13_RHEUMA), download from https://storage.googleapis.com/finngen-public-data-r9/summary_stats/finngen_R9_M13_RHEUMA.gz | European |

Supplementary Table 2. Genetic variants that preferentially satisfied instrument assumptions of the MR analysis

| Exposure | Outcome | SNP | effect_allele | other_allele | pval.exposure |
| --- | --- | --- | --- | --- | --- |
| EMs | ukb-d-M06 |  |  |  |  |
|  |  | rs10022464 | A | G | 3.15E-10 |
|  |  | rs10067198 | A | G | 2.425E-09 |
|  |  | rs10491147 | C | G | 9.58E-12 |
|  |  | rs10758664 | T | G | 1.947E-10 |
|  |  | rs10759351 | T | C | 2.286E-09 |
|  |  | rs10773149 | T | G | 3.287E-08 |
|  |  | rs10836159 | T | C | 4.977E-08 |
|  |  | rs11062038 | T | C | 4.373E-09 |
|  |  | rs111228597 | T | C | 1.678E-08 |
|  |  | rs11125864 | T | G | 9.922E-09 |
|  |  | rs111315673 | T | G | 8.327E-09 |
|  |  | rs111699929 | A | C | 1.435E-08 |
|  |  | rs111827656 | A | C | 3.737E-08 |
|  |  | rs112222992 | T | C | 7.84E-09 |
|  |  | rs112701277 | T | C | 1.911E-10 |
|  |  | rs113593574 | T | C | 3.109E-10 |
|  |  | rs115527226 | A | G | 3.304E-08 |
|  |  | rs11592820 | A | T | 3.597E-09 |
|  |  | rs116183328 | A | G | 6.984E-14 |
|  |  | rs116329098 | A | C | 2.847E-10 |
|  |  | rs11672757 | A | G | 7.917E-12 |
|  |  | rs116859443 | T | C | 4.485E-08 |
|  |  | rs11701433 | T | C | 9.072E-09 |
|  |  | rs118008737 | T | C | 2.438E-10 |
|  |  | rs118157238 | A | G | 5.32E-09 |
|  |  | rs11994887 | T | C | 1.64E-08 |
|  |  | rs12185655 | A | G | 1.44E-08 |
|  |  | rs12410893 | A | G | 1.687E-08 |
|  |  | rs12672158 | A | G | 2.009E-09 |
|  |  | rs13035259 | A | C | 3.728E-12 |
|  |  | rs13227807 | T | C | 9.577E-09 |
|  |  | rs13426089 | T | C | 2.358E-11 |
|  |  | rs1350674 | T | C | 1.752E-08 |
|  |  | rs138845060 | T | C | 6.014E-10 |
|  |  | rs1394619 | A | G | 1.313E-08 |
|  |  | rs139615317 | T | C | 8.265E-09 |
|  |  | rs141107755 | A | G | 4.966E-13 |
|  |  | rs141717433 | T | C | 1.43E-10 |
|  |  | rs142346408 | A | G | 3.03E-08 |
|  |  | rs142409403 | A | G | 2.013E-09 |
|  |  | rs142421421 | A | G | 5.376E-11 |
|  |  | rs145097158 | T | G | 3.182E-09 |
|  |  | rs145171510 | C | G | 1.057E-09 |
|  |  | rs145910477 | T | G | 1.842E-08 |
|  |  | rs149036775 | T | C | 1.606E-13 |
|  |  | rs150789134 | A | G | 4.701E-08 |
|  |  | rs1667351 | A | T | 2.036E-08 |
|  |  | rs16869446 | T | C | 4.014E-08 |
|  |  | rs17061886 | T | C | 4.748E-08 |
|  |  | rs17311489 | A | G | 3.51E-09 |
|  |  | rs17365446 | C | G | 1.804E-13 |
|  |  | rs17406405 | A | G | 1.628E-11 |
|  |  | rs1771178 | A | G | 2.838E-08 |
|  |  | rs17835714 | T | C | 3.632E-08 |
|  |  | rs180676 | A | G | 2.915E-11 |
|  |  | rs182324074 | A | G | 6.866E-09 |
|  |  | rs182838373 | C | G | 5.639E-09 |
|  |  | rs182867533 | A | T | 1.251E-08 |
|  |  | rs183873688 | C | G | 4.118E-08 |
|  |  | rs189094443 | T | C | 1.212E-09 |
|  |  | rs194134 | T | C | 1.047E-08 |
|  |  | rs201878599 | C | G | 2.079E-09 |
|  |  | rs2341389 | T | C | 2.609E-09 |
|  |  | rs2408211 | T | C | 1.617E-08 |
|  |  | rs2440028 | A | G | 1.084E-09 |
|  |  | rs2895311 | A | G | 1.328E-09 |
|  |  | rs3013455 | T | C | 5.552E-11 |
|  |  | rs34261801 | T | C | 3.557E-08 |
|  |  | rs34525429 | C | G | 1.793E-09 |
|  |  | rs34642578 | T | C | 2.153E-08 |
|  |  | rs34929394 | C | G | 1.776E-08 |
|  |  | rs35343740 | T | C | 1.113E-09 |
|  |  | rs379949 | A | G | 3.211E-08 |
|  |  | rs4639295 | A | G | 2.079E-08 |
|  |  | rs4772856 | A | G | 2.593E-09 |
|  |  | rs4811012 | T | C | 0.000000013 |
|  |  | rs4819527 | T | C | 1.106E-08 |
|  |  | rs5007400 | A | T | 5.657E-09 |
|  |  | rs551209543 | C | G | 2.538E-08 |
|  |  | rs55747751 | A | G | 2.175E-09 |
|  |  | rs56078105 | T | C | 2.932E-08 |
|  |  | rs57590076 | T | C | 3.085E-09 |
|  |  | rs586913 | T | C | 1.36E-08 |
|  |  | rs61176465 | T | C | 2.57E-10 |
|  |  | rs61910038 | T | C | 4.204E-08 |
|  |  | rs6505587 | T | C | 1.26E-10 |
|  |  | rs658289 | T | C | 1.372E-08 |
|  |  | rs6661757 | T | C | 2.292E-09 |
|  |  | rs6665821 | A | T | 3.206E-08 |
|  |  | rs6812266 | C | G | 3.403E-08 |
|  |  | rs72679720 | T | C | 4.638E-08 |
|  |  | rs72782359 | T | C | 5.637E-09 |
|  |  | rs72787757 | T | C | 2.618E-09 |
|  |  | rs72882089 | T | C | 1.437E-08 |
|  |  | rs72981722 | A | G | 1.015E-08 |
|  |  | rs73022571 | A | G | 4.991E-08 |
|  |  | rs73114447 | A | G | 2.804E-08 |
|  |  | rs73276486 | A | G | 1.475E-09 |
|  |  | rs73823391 | T | C | 1.238E-08 |
|  |  | rs74072326 | A | G | 1.265E-08 |
|  |  | rs74342842 | A | G | 3.724E-08 |
|  |  | rs74869083 | T | C | 1.56E-11 |
|  |  | rs76969132 | A | G | 2.095E-08 |
|  |  | rs7744271 | T | C | 3.134E-09 |
|  |  | rs78770528 | T | C | 3.88E-08 |
|  |  | rs79319004 | A | T | 4.009E-08 |
|  |  | rs79774159 | T | C | 6.576E-10 |
|  |  | rs79829688 | T | G | 1.835E-08 |
|  |  | rs80103564 | A | T | 1.396E-09 |
|  |  | rs80103753 | C | G | 4.299E-08 |
|  |  | rs9369929 | T | C | 5.613E-09 |
|  |  | rs9439538 | T | C | 9.509E-09 |
|  |  | rs9559006 | T | G | 5.802E-09 |
|  |  | rs9788865 | T | C | 2.006E-08 |
| EMs | ukb-b-11874 |  |  |  |  |
|  |  | rs10759351 | T | C | 2.286E-09 |
|  |  | rs10836159 | T | C | 4.977E-08 |
|  |  | rs11062038 | T | C | 4.373E-09 |
|  |  | rs12185655 | A | G | 1.44E-08 |
|  |  | rs12410893 | A | G | 1.687E-08 |
|  |  | rs12672158 | A | G | 2.009E-09 |
|  |  | rs2341389 | T | C | 2.609E-09 |
|  |  | rs34261801 | T | C | 3.557E-08 |
|  |  | rs34525429 | C | G | 1.793E-09 |
|  |  | rs379949 | A | G | 3.211E-08 |
|  |  | rs4639295 | A | G | 2.079E-08 |
|  |  | rs4772856 | A | G | 2.593E-09 |
|  |  | rs4819527 | T | C | 1.106E-08 |
|  |  | rs57590076 | T | C | 3.085E-09 |
|  |  | rs586913 | T | C | 1.36E-08 |
|  |  | rs6661757 | T | C | 2.292E-09 |
|  |  | rs72882089 | T | C | 1.437E-08 |
|  |  | rs79319004 | A | T | 4.009E-08 |
|  |  | rs9369929 | T | C | 5.613E-09 |
|  |  | rs9439538 | T | C | 9.509E-09 |
|  |  | rs9788865 | T | C | 2.006E-08 |
| EMs | ukb-b-9125 |  |  |  |  |
|  |  | rs12410893 | A | G | 1.687E-08 |
|  |  | rs3013455 | T | C | 5.552E-11 |
|  |  | rs57590076 | T | C | 3.085E-09 |
|  |  | rs6661757 | T | C | 2.292E-09 |
|  |  | rs72679720 | T | C | 4.638E-08 |
|  |  | rs72882089 | T | C | 1.437E-08 |
|  |  | rs9439538 | T | C | 9.509E-09 |
| EMs | ukb-a-105 |  |  |  |  |
|  |  | rs12410893 | A | G | 1.687E-08 |
|  |  | rs56078105 | T | C | 2.932E-08 |
|  |  | rs6661757 | T | C | 2.292E-09 |
|  |  | rs72882089 | T | C | 1.437E-08 |
|  |  | rs9439538 | T | C | 9.509E-09 |
| EMs | ukb-d-M06 |  |  |  |  |
|  |  | rs10022464 | A | G | 3.15E-10 |
|  |  | rs10067198 | A | G | 2.425E-09 |
|  |  | rs10491147 | C | G | 9.58E-12 |
|  |  | rs10758664 | T | G | 1.947E-10 |
|  |  | rs10759351 | T | C | 2.286E-09 |
|  |  | rs10773149 | T | G | 3.287E-08 |
|  |  | rs10836159 | T | C | 4.977E-08 |
|  |  | rs11062038 | T | C | 4.373E-09 |
|  |  | rs111228597 | T | C | 1.678E-08 |
|  |  | rs11125864 | T | G | 9.922E-09 |
|  |  | rs111315673 | T | G | 8.327E-09 |
|  |  | rs111699929 | A | C | 1.435E-08 |
|  |  | rs111827656 | A | C | 3.737E-08 |
|  |  | rs112222992 | T | C | 7.84E-09 |
|  |  | rs112701277 | T | C | 1.911E-10 |
|  |  | rs113593574 | T | C | 3.109E-10 |
|  |  | rs115527226 | A | G | 3.304E-08 |
|  |  | rs11592820 | A | T | 3.597E-09 |
|  |  | rs116183328 | A | G | 6.984E-14 |
|  |  | rs116329098 | A | C | 2.847E-10 |
|  |  | rs11672757 | A | G | 7.917E-12 |
|  |  | rs116859443 | T | C | 4.485E-08 |
|  |  | rs11701433 | T | C | 9.072E-09 |
|  |  | rs118008737 | T | C | 2.438E-10 |
|  |  | rs118157238 | A | G | 5.32E-09 |
|  |  | rs11994887 | T | C | 1.64E-08 |
|  |  | rs12185655 | A | G | 1.44E-08 |
|  |  | rs12410893 | A | G | 1.687E-08 |
|  |  | rs12672158 | A | G | 2.009E-09 |
|  |  | rs13035259 | A | C | 3.728E-12 |
|  |  | rs13227807 | T | C | 9.577E-09 |
|  |  | rs13426089 | T | C | 2.358E-11 |
|  |  | rs1350674 | T | C | 1.752E-08 |
|  |  | rs138845060 | T | C | 6.014E-10 |
|  |  | rs1394619 | A | G | 1.313E-08 |
|  |  | rs139615317 | T | C | 8.265E-09 |
|  |  | rs141107755 | A | G | 4.966E-13 |
|  |  | rs141717433 | T | C | 1.43E-10 |
|  |  | rs142346408 | A | G | 3.03E-08 |
|  |  | rs142409403 | A | G | 2.013E-09 |
|  |  | rs142421421 | A | G | 5.376E-11 |
|  |  | rs145097158 | T | G | 3.182E-09 |
|  |  | rs145171510 | C | G | 1.057E-09 |
|  |  | rs145910477 | T | G | 1.842E-08 |
|  |  | rs149036775 | T | C | 1.606E-13 |
|  |  | rs150789134 | A | G | 4.701E-08 |
|  |  | rs1667351 | A | T | 2.036E-08 |
|  |  | rs16869446 | T | C | 4.014E-08 |
|  |  | rs17061886 | T | C | 4.748E-08 |
|  |  | rs17311489 | A | G | 3.51E-09 |
|  |  | rs17365446 | C | G | 1.804E-13 |
|  |  | rs17406405 | A | G | 1.628E-11 |
|  |  | rs1771178 | A | G | 2.838E-08 |
|  |  | rs17835714 | T | C | 3.632E-08 |
|  |  | rs180676 | A | G | 2.915E-11 |
|  |  | rs182324074 | A | G | 6.866E-09 |
|  |  | rs182838373 | C | G | 5.639E-09 |
|  |  | rs182867533 | A | T | 1.251E-08 |
|  |  | rs183873688 | C | G | 4.118E-08 |
|  |  | rs189094443 | T | C | 1.212E-09 |
|  |  | rs194134 | T | C | 1.047E-08 |
|  |  | rs201878599 | C | G | 2.079E-09 |
|  |  | rs2341389 | T | C | 2.609E-09 |
|  |  | rs2408211 | T | C | 1.617E-08 |
|  |  | rs2440028 | A | G | 1.084E-09 |
|  |  | rs2895311 | A | G | 1.328E-09 |
|  |  | rs3013455 | T | C | 5.552E-11 |
|  |  | rs34261801 | T | C | 3.557E-08 |
|  |  | rs34525429 | C | G | 1.793E-09 |
|  |  | rs34642578 | T | C | 2.153E-08 |
|  |  | rs34929394 | C | G | 1.776E-08 |
|  |  | rs35343740 | T | C | 1.113E-09 |
|  |  | rs379949 | A | G | 3.211E-08 |
|  |  | rs4639295 | A | G | 2.079E-08 |
|  |  | rs4772856 | A | G | 2.593E-09 |
|  |  | rs4811012 | T | C | 0.000000013 |
|  |  | rs4819527 | T | C | 1.106E-08 |
|  |  | rs5007400 | A | T | 5.657E-09 |
|  |  | rs551209543 | C | G | 2.538E-08 |
|  |  | rs55747751 | A | G | 2.175E-09 |
|  |  | rs56078105 | T | C | 2.932E-08 |
|  |  | rs57590076 | T | C | 3.085E-09 |
|  |  | rs586913 | T | C | 1.36E-08 |
|  |  | rs61176465 | T | C | 2.57E-10 |
|  |  | rs61910038 | T | C | 4.204E-08 |
|  |  | rs6505587 | T | C | 1.26E-10 |
|  |  | rs658289 | T | C | 1.372E-08 |
|  |  | rs6661757 | T | C | 2.292E-09 |
|  |  | rs6665821 | A | T | 3.206E-08 |
|  |  | rs6812266 | C | G | 3.403E-08 |
|  |  | rs72679720 | T | C | 4.638E-08 |
|  |  | rs72782359 | T | C | 5.637E-09 |
|  |  | rs72787757 | T | C | 2.618E-09 |
|  |  | rs72882089 | T | C | 1.437E-08 |
|  |  | rs72981722 | A | G | 1.015E-08 |
|  |  | rs73022571 | A | G | 4.991E-08 |
|  |  | rs73114447 | A | G | 2.804E-08 |
|  |  | rs73276486 | A | G | 1.475E-09 |
|  |  | rs73823391 | T | C | 1.238E-08 |
|  |  | rs74072326 | A | G | 1.265E-08 |
|  |  | rs74342842 | A | G | 3.724E-08 |
|  |  | rs74869083 | T | C | 1.56E-11 |
|  |  | rs76969132 | A | G | 2.095E-08 |
|  |  | rs7744271 | T | C | 3.134E-09 |
|  |  | rs78770528 | T | C | 3.88E-08 |
|  |  | rs79319004 | A | T | 4.009E-08 |
|  |  | rs79774159 | T | C | 6.576E-10 |
|  |  | rs79829688 | T | G | 1.835E-08 |
|  |  | rs80103564 | A | T | 1.396E-09 |
|  |  | rs80103753 | C | G | 4.299E-08 |
|  |  | rs9369929 | T | C | 5.613E-09 |
|  |  | rs9439538 | T | C | 9.509E-09 |
|  |  | rs9559006 | T | G | 5.802E-09 |
|  |  | rs9788865 | T | C | 2.006E-08 |

Supplementary Table 3. Characteristics of patients with rheumatoid arthritis from 1999 to 2006 NHANES, weighted.

| **Variable** | **Total** | **Rheumatoid Arthritis** | | **P value** |
| --- | --- | --- | --- | --- |
|  |  | **No** | **Yes** |  |
| **Overall, n** | 2591 | 2496 | 95 |  |
| Age, years, mean ± SD | 37.38 (0.26) | 37.21 (0.26) | 42.44 (0.90) | ***< 0.0001*** |
| BMI, kg/m^2^, mean ± SD | 27.53 (0.22) | 27.43 (0.22) | 30.34 (0.83) | ***< 0.001*** |
| Race, n (%) |  |  |  | 0.11 |
| Non-Hispanic White | 1361 (73.83) | 1320 (74.13) | 41 (64.63) |  |
| Non-Hispanic Black | 496 (9.87) | 469 (9.64) | 27 (17.12) |  |
| Mexican American | 524 (6.70) | 507 (6.71) | 17 (6.40) |  |
| Other Hispanic | 122 (5.61) | 117 (5.63) | 5 (4.99) |  |
| Other Race-Including Multi-Racial | 88 (3.98) | 83 (3.89) | 5 (6.86) |  |
| Marital status, n (%) |  |  |  | ***0.01*** |
| Married/Living with partner | 1629 (66.15) | 1576 (66.09) | 53 (67.88) |  |
| Never married | 551 (18.54) | 540 (18.89) | 11 (7.91) |  |
| Divorced/Separated/Widowed | 411 (15.31) | 380 (15.02) | 31 (24.20) |  |
| Education level, n (%) |  |  |  | ***0.02*** |
| College graduate or above | 683 (31.14) | 669 (31.64) | 14 (16.00) |  |
| Some college or AA degree | 943 (37.24) | 904 (37.06) | 39 (42.79) |  |
| High school/GED/Less than 11th grade | 965 (31.62) | 923 (31.30) | 42 (41.21) |  |
| Smoking, n (%) |  |  |  | ***0.004*** |
| No | 1582 (58.53) | 1537 (59.08) | 45 (41.67) |  |
| Yes | 1009 (41.47) | 959 (40.92) | 50 (58.33) |  |
| Alcohol status, n (%) |  |  |  | ***< 0.001*** |
| Never | 371 (12.24) | 357 (12.13) | 14 (15.60) |  |
| Former | 342 (12.09) | 316 (11.60) | 26 (27.20) |  |
| Mild/Moderate | 1299 (53.15) | 1263 (53.60) | 36 (39.41) |  |
| Heavy | 579 (22.52) | 560 (22.67) | 19 (17.78) |  |
| Income to poverty ratio, mean ± SD | 3.17 (0.06) | 3.18 (0.06) | 3.03 (0.17) | 0.39 |
| Physical activity, MET-h/week, mean ± SD | 825.65 (38.35) | 817.94 (37.86) | 1061.73 (179.66) | 0.17 |
| SUA, μmol/L, mean ± SD | 264.83 (1.70) | 264.38 (1.66) | 278.35 (8.86) | 0.11 |
| Chronic kidney disease, n (%) |  |  |  | ***0.01*** |
| No | 2379 (92.83) | 2298 (93.08) | 81 (85.35) |  |
| Yes | 212 (7.17) | 198 (6.92) | 14 (14.65) |  |
| Diabetes mellitus, n (%) |  |  |  | 0.10 |
| No | 2480 (96.69) | 2393 (96.79) | 87 (93.49) |  |
| Yes | 111 (3.31) | 103 (3.21) | 8 (6.51) |  |
| Hyperlipidemia, n (%) |  |  |  | ***0.01*** |
| No | 930 (36.77) | 908 (37.29) | 22 (20.81) |  |
| Yes | 1661 (63.23) | 1588 (62.71) | 73 (79.19) |  |
| Hypertension, n (%) |  |  |  | ***< 0.001*** |
| No | 2071 (80.74) | 2010 (81.23) | 61 (65.82) |  |
| Yes | 520 (19.26) | 486 (18.77) | 34 (34.18) |  |
| Endometriosis, n (%) |  |  |  | ***0.01*** |
| No | 2399 (91.23) | 2319 (91.52) | 80 (82.44) |  |
| Yes | 192 (8.77) | 177 (8.48) | 15 (17.56) |  |

*Notes:* * All estimates accounted for sample weights and complex survey designs, and percentages were adjusted for survey weights of NHANES. Abbreviation: BMI, body mass index; GED, general educational development; NHANES, National Health and Nutrition Examination Survey; SD, standard deviation; SUA, serum uric acid; *p* value in bold indicates statistical significance.

Supplementary Table 4. The assessments of Horizontal pleiotropy

| Exposure | Outcome | No of IVs | Egger Intercept | SE | P value |
| --- | --- | --- | --- | --- | --- |
| EMs | finn-b-M13_RHEUMA | 114 | -1.69E-05 | 5.34E-05 | 0.752674659 |
| EMs | ukb-a-105 | 5 | -0.000397005 | 0.000451075 | 0.443592681 |
| EMs | ukb-b-9125 | 7 | 0.001395308 | 0.000978758 | 0.213311158 |
| EMs | ukb-b-11874 | 21 | 0.001001184 | 0.000491963 | 0.056036528 |
| EMs | ukb-d-M06 | 114 | 1.01E-06 | 5.13E-05 | 0.984368283 |

Supplementary Table 5. The assessments of heterogeneity

| Exposure | Outcome | Q | Q_df | P value for Cochran's Q test |
| --- | --- | --- | --- | --- |
| EMs | finn-b-M13_RHEUMA | 111.3818233 | 113 | 0.525411316 |
| EMs | ukb-a-105 | 3.160502638 | 4 | 0.531333968 |
| EMs | ukb-b-9125 | 3.623351396 | 6 | 0.727492533 |
| EMs | ukb-b-11874 | 23.13188837 | 20 | 0.282365043 |
| EMs | ukb-d-M06 | 109.0444711 | 113 | 0.587748462 |

Supplementary Table 6. The assessments of direction of causal relationship

| Exposure | Outcome | Correct Causal direction | P value for MR-steiger |
| --- | --- | --- | --- |
| EMs | finn-b-M13_RHEUMA | TRUE | 4.61E-161 |
| EMs | ukb-a-105 | TRUE | 5.28E-08 |
| EMs | ukb-b-9125 | TRUE | 1.12E-14 |
| EMs | ukb-b-11874 | TRUE | 5.48E-34 |
| EMs | ukb-d-M06 | TRUE | 3.43E-162 |
